# BF_3_·Et_2_O Catalysed 4-Aryl-3-phenyl-benzopyrones, Pro-SERMs, and Their Characterization

**DOI:** 10.1155/2015/527159

**Published:** 2015-09-01

**Authors:** Ambika Srivastava, Pooja Singh, Rajesh Kumar

**Affiliations:** Department of Chemistry, Centre of Advanced Study, Faculty of Science, Banaras Hindu University, Varanasi 221005, India

## Abstract

We have synthesized the novel 4-(4-hydroxy-benzyl)-3-phenyl-chromen-2-one which is a precursor of SERMs with a smaller number of steps and good yield. Two methodologies for the synthesis have been worked out. Anhydrous BF_3_·Et_2_O catalyzed reaction was found to be selective for product formation while anhydrous AlCl_3_, FeCl_3_, and SnCl_4_ catalyzed ones were nonselective.

## 1. Introduction

**Figure 1:**
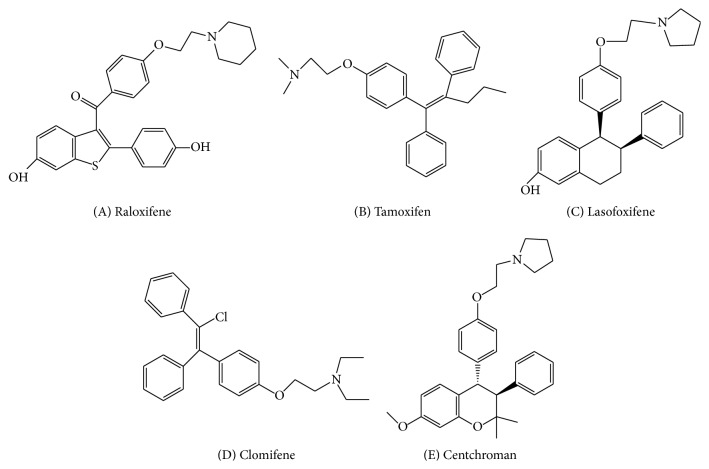
Examples of biologically active heterocyclic frameworks.

Activity of estrogen receptor can be controlled by a class of compounds which is called selective estrogen receptor modulators (SERMs). The modulators have a distinctive feature in different individual tissues by which they can inhibit or stimulate or selectively suppress or excite estrogen-like behavior in different tissues. The structures of few biologically vital SERMs are shown in Figure 1 in which compound A is a polyhydroxy phenyl benzothiophene which has low physiological response when combined with the receptor estrogen in gnawing uterus [1]. Compound A was initially known as Ly156758/keoxifen and its advancement has been stopped for improved action for treatment of breast cancer [2] due to less bioavailability than the required essential dose [3]. But the concept of SERM was shown by compound A due to sustainable property of bone density [4, 5] with restriction in mammary carcinogenesis in rat [6, 7]. Studies on compound A reveal the reduction of risk of osteoporosis [8] and breast cancer in women after menopause [9]. Compound B is a nonhydroxy and typical model compound for SERM which was used clinically for the occupational therapy of breast cancer [10, 11] with the maintained density in bone of women after menopause [12]. The drawback of the treatment by compound B was the increased possibilities of endometrial cancer [13]. Hence, it is clear that there are various factors which are responsible for estrogenic and antiestrogenic properties of SERM complexes and could be useful in improving targeted therapeutic agents.

Coumarin and its derivatives are important compounds due to their presence in numerous natural products along with their wide ranging applications as drugs, pharmaceuticals, and SERMs. Coumarin based selective estrogen receptor modulators (SERMs) and coumarin-estrogen conjugates have been described as potential anti-breast-cancer agents. Thus, coumarin derivatives acting as SERMs either stimulate or inhibit the estrogen action, thereby generating the possibility of curing estrogen related problems. Coumarins and their derivatives are common in nature [14–18]; among them the 4-substituted coumarins were identified as anticancer and anti-HIV-1 molecules [19, 20].

**Scheme 1:**
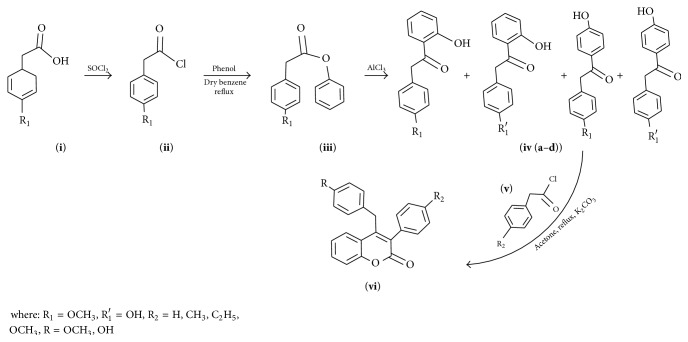
Route 1 for the synthesis of coumarin based SERM's precursors.

**Scheme 2:**
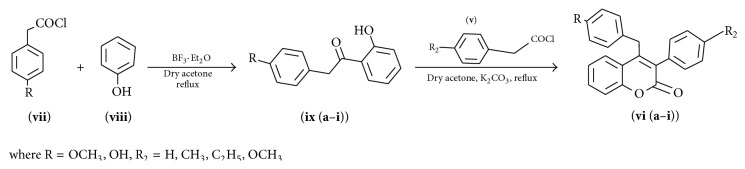
Route 2 for the synthesis of coumarin based SERM's precursors.

Among the oxygen heterocycles coumarins are one of them which are present in various naturally [21, 22] occurring motifs. Due to comprehensive and inexhaustible performance of coumarin in biological activities [23–33] such as anti-HIV [34–37], anticoagulation [38], antibiotic [39–42], anticancer [43, 44], anti-inflammatory [45, 46], antioxidant [47–49], antitumor [50–52], antiviral [53], antihypertensive, and antimicrobial activity its chemistry grew up widely. Among the nuclear hormone receptor modulators,* namely*, SERMs, PRMs, and SARMs [54–58], coumarins are also identified with a similar kind of properties. Among the coumarin derivatives more attention is given to 4-substituted coumarins, but there are very few methods known for synthesis. Route 1 (Scheme 1) to coumarins incorporates Pechmann [59, 60], Knoevenagel [61–64], Reformatsky [61–64], Perkin [65], and Wittig [66] condensation reactions. To make these reactions efficacious, several variations in terms of catalyst and reaction conditions have been done. However, the route 1 methodology suffers from laborious multistep procedures, long reaction time, high reaction temperature, nonselectivity, and waste problem. To overcome these, a facile two-step synthesis of 4-aryl-3-phenyl-coumarin-2-one has been reported as shown in Scheme 2, which would be helpful in designing novel SERMs.

## 2. Results and Discussion

Condensation reactions have been amongst the most useful routes for the synthesis of these compounds, particularly catalyzed by Lewis acids. In Scheme 1, 4-methoxy phenyl acetic acid and phenol were taken as starting material. In the first step, acyl chloride of acid was prepared, where the yield of phenyl acyl chloride obtained was 50%. Further esterification led to some good yield, but the yield was very poorly shed down to 10% with next step reaction, that is, Fries rearrangement. The reaction of ester and AlCl_3_ at 145°C led to four products, of which only two (**iv**-**a, iv**-**b**) were important for synthesis purpose. Fries rearrangement with AlCl_3_ has no selectivity and gave four products with almost 10% yield, which were separated chromatographically. Then cyclization with phenyl acetyl chloride was carried out with anhydrous K_2_CO_3_ in dry acetone. There were some shortcomings like low reaction yields and nonselectivity of reaction/more byproduct formation/low atom economic reactions. Hence, the nonselectivity of reactions (via Scheme 1) and low atom economy demanded the search for a simple, short, and high-yielding alternate process to synthesize substituted coumarin based SERMs precursors.

**Figure 2:**
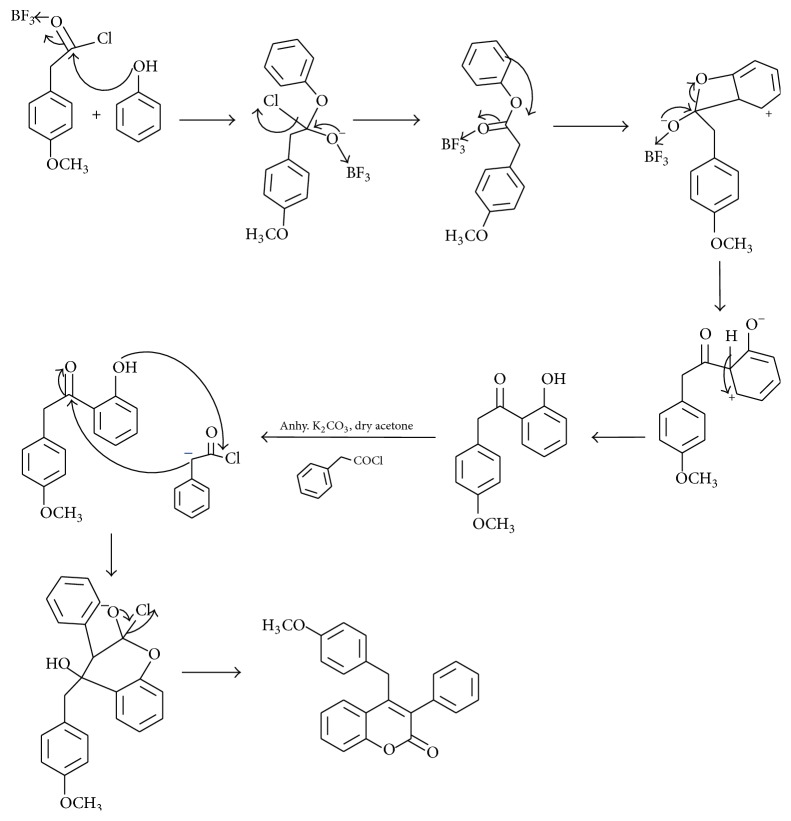
Probable mechanism related to Scheme 2.

To decrease the product loss and number of steps, the synthetic strategy was modified and Scheme 2 route was selected in which 4-substituted phenyl acetic acid and substituted phenol were used as starting material and reaction was catalyzed by BF_3_·Et_2_O which was found to be a very efficient catalyst. In this report, a facile and high-yielding protocol for diverse SERMs precursors through synthesis of functionalized benzylic ketone and further intermolecular cyclization using substituted phenyl acetyl chloride with dry acetone and potassium carbonate under reflux condition has been described. Further, to our ongoing research on novel synthetic methodologies for SERMs precursors synthesis, we commenced our synthetic strategy with environmentally benign phenol, which on coupling with different phenyl acetyl chlorides including p-anisole acetyl chloride, p-phenyl acetyl chloride, and p-hydroxy phenyl acetyl chloride afforded substituted benzylic ketones in good yields. The substituted benzylic ketones (**ix (a**–**i)**) on further treatment with substituted phenyl acetyl chloride in the presence of K_2_CO_3_ and dry acetone led to the formation of various substituted SERMs precursors (4-benzyl-3-phenyl coumarin) (**vi (a**–**i)**), in good yields (Scheme 2). Thus, the synthesis of substituted SERMs precursor (4-benzyl-3-phenyl coumarin) was achieved in two steps. Acetylation was regioselective and occurred at ortho position which was the major reaction product. Thus, in just one step, phenol was esterified and the ester readily rearranged to give 4-methoxy phenyl acetyl group at ortho position of phenol. This stage product was achieved by Scheme 1 after 3 steps with low atom economy and many undesirable products. The intermediate ester (Scheme 2) could not be isolated since BF_3_·Et_2_O readily rearranged it to ortho substituted phenol. Thus, the two-step process was reduced to one step, the probable mechanism of which has been given in Figure 2.

In our early attempts, to synthesize the coumarin based SERMs precursors, we were not successful in converting the reactants to products without the catalyst (BF_3_·Et_2_O). The anhydrous AlCl_3_, FeCl_3_, and SnCl_4_ were not able to give the desired intermediate selectively in quantitative yield. This was possibly due to poor Lewis acid character of AlCl_3_, FeCl_3_, and SnCl_4_ compared to BF_3_. The reaction was investigated carefully and it was observed that the intermediate (benzylic ketones (**ix (a**–**i)**)) formed after the coupling of phenol with substituted phenyl acetyl chloride was sufficiently stable and could be isolated. In the second step intermolecular cyclization was carried out with substituted phenyl acetyl chloride and a base (anhydrous K_2_CO_3_). The desired product (**vi-e**) was characterized by ^1^H NMR (Figure S6(a) in Supplementary Material available online at http://dx.doi.org/10.1155/2015/527159), which contains additional peaks at *δ* 6.79 and 6.98 due to benzylic proton and at *δ* 7.2 and 7.3 due to phenylic protons and one signal at *δ* 7.15 was due to proton at para position in the phenyl ring. The rest of the protons were the same as in the precursor, that is, ortho substituted phenol (**iv-a**).

^13^C NMR (Figure S6(b)) also confirmed the formation of 4-(4-hydroxy-benzyl)-3-phenyl-chromen-2-one; peaks at 119.60, 126.40, 128.38, 129.53, 134.05, and 161.22 show six different types of carbons which are present in 4-aryl-3-phenyl-benzopyrone in addition to the carbons already present in the starting, that is, 2-(4-hydroxy-phenyl)-1-(2-hydroxy phenyl)-ethanone. FTIR spectrum also confirmed the formation of lactone ring; that is, the cyclized product shows carbonyl absorption at a higher wavenumber, that is, at 1707 cm^−1^ (Figure S6(c)), while it was 1633 cm^−1^ in the 2-(4-hydroxy-phenyl)-1-(2-hydroxy-phenyl)-ethanone (Figure S2(a)). Mass spectroscopy shows (m + 1) peak at 343 while the molecular weight of (**vi-e**) is 342 (Figure S6(d)).

**Figure 3:**
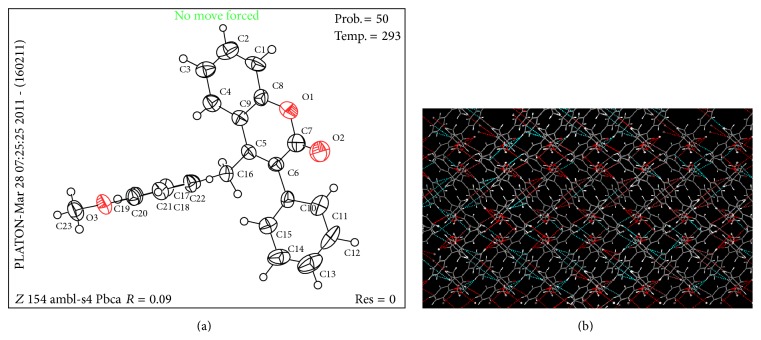
(a) ORTEP/PLATON structure of (**vi**-**e**). (b) Packing structure of (**vi**-**e**) showing Z-like packing.

Finally the single crystal diffraction studies showed the space orientation (Figures 3(a) and 3(b)), bond lengths, and bond angles regarding the crystal structure (Table 1). The structure reflects that the coumarin ring is planar, phenyl ring which is attached at position 3 is slightly out of plane, and substituted benzylic group is perpendicular to the ring coumarin (Figure 3(a)). Compound (**vi-e**) exhibited “Z-” like packing diagram (Figure 3(b)).

This new procedure allows facile introduction of substituents at position 4 of the 4-(4-substituted-benzyl)-3-phenyl-chromen-2-one skeleton and gives the flexibility for the construction of novel precursors.

Various derivatives have been prepared with para substituted benzyl chloride with hydroxyl, methoxy, acetoxy, methyl, and ethyl groups as shown in Table 2. All the derivatives have been prepared smoothly under the same reaction conditions. The reactions are simple, easy to handle, and feasible and have simple workup procedures.

After the establishment of the protocol for the synthesis of substituted SERMs precursors (4-benzyl-3-phenyl coumarins), we shifted our focus towards the role of solvents like CH_2_Cl_2_, CHCl_3_, acetone, and toluene upon yield and the reaction time. The results illustrated that the reaction in toluene did not give the desired precursors, whereas the reaction in CHCl_3_ was slow and the yield was low. However, for this cyclization, CH_2_Cl_2_ was found to be good in terms of yield and handling but took a slightly longer time to afford the products. Eventually, acetone appeared as a solvent of choice for intermolecular cyclization in very good yield. Intermolecular cyclization was greatly influenced by the base used; therefore, to find out the appropriate base, we examined K_2_CO_3_ and triethylamine in the intermolecular cyclization reaction of (**ix-a**) with (**v**) and found that the reaction in the presence of K_2_CO_3_ afforded the cyclized product (**vi-a**) in 74% yield after 7 h, whereas triethylamine gave this product in 57% yield. We believe that potassium carbonate may be more dissociated in aprotic polar solvents and consequently proved to be more reactive.

## 3. Conclusion

In conclusion, a simple, efficient, and novel method has been developed for an easy access to synthesis of the 4-(4-hydroxy-benzyl)-3-phenyl-chromen-2-one via Scheme 2 and this has been supported by ^1^H NMR, FTIR, ^13^C NMR, mass spectroscopy, and single crystal X-ray data analysis. Synthetic pathway with just 2 steps proved to be the best with less side reactions and greater yield. Thus, the number of steps has been decreased and the yield was increased. Herein we reported some precursors of coumarin based SERMs which could be useful in designing new SERMs. The pure products were obtained by column chromatography. This methodology presents several advantages including (a) mild reaction conditions, (b) simple workup procedure, (c) moderately high yields of the desired products, (d) the selectivity of the product, and finally (e) economic availability of the reagents making the whole process simple and feasible. Efforts to extend the span of the procedure on SERMs are under progress in our laboratory.

## 4. Experimental Section

### 4.1. General Methods

All the required chemicals are purchased since they are commercially available and used as received without further purification. Commercially available acetone and benzene were further purified and dried following the known procedure. Thin-layer chromatography (TLC) was performed using silica gel 60 F254 precoated plates. Column chromatography was carried out on silica gel 60 (100–200 mesh). Infrared (FTIR) spectra were recorded in KBr, and wavelengths (*ν*) have been reported in cm^−1^; ^1^H and ^13^C NMR spectra were recorded on NMR spectrometers operating at 300 and 75.5 MHz, respectively. Chemical shifts (*δ*) were given in parts per million (ppm) using the residue solvent peaks as reference relative to TMS. *J* values have been given in Hz. Mass spectra were recorded using electrospray ionization (ESI) mass spectrometer. The melting points were taken in open capillary and uncorrected.

#### 4.1.1. Procedure for Scheme 1

*Compound (* ***ii***). To a solution of 4-methoxy phenyl acetic acid (42.5 g, 0.25 mol) in dry benzene (50 mL) was added thionyl chloride (30 mL, 0.25 mol) dropwise with syringe. After the reaction was complete, the reaction mixture was distilled to remove excess thionyl chloride and the solvent benzene. Brown colored liquid was obtained. Yield:- 50%, ^1^H NMR- (300 MHz, CDCl_3_): *δ* 3.77 (s, 3H, -CH_3_), 4.20 (s, 2H, -CH_2_), 6.68 (d, *J* = 7.8 Hz, 2H, Ar-H), 7.10 (d, *J* = 7.8 Hz, 2H, Ar-H).

*Compound (* ***iii***). A solution of p-methoxy phenyl acetyl chloride (24 g, 0.13 mol) and phenol (12.2 mL, 0.13 mol) in dry benzene (63 mL) was refluxed for 21 h, till the reaction was complete as monitored by TLC. Then the reaction mixture was washed with 5% aqueous NaOH to remove excess unreacted phenol and then washed with water three times and dried over anhydrous Na_2_SO_4_ and concentrated over vacuum. Orange colored liquid compound was obtained. Yield:- 70%  ^1^H NMR- (300 MHz, CDCl_3_): *δ* 3.54 (s, 2H, -CH_2_), 3.77 (s, 3H, -CH_3_), 6.70 (d, *J* = 7.6 Hz, 2H, -Ar-H), 7.10 (d, *J* = 7.8 Hz, 2H, -Ar-H), 7.23 (m, 3H, -Ar-H), 7.35 (t, *J* = 7.9 Hz, 2H, -Ar-H); FTIR (KBr, cm^−1^): 2937, 2837, 1755, 1600, 1513, 1300, 1248, 1125, 814 (Figure S1(a & b); *m*/*z* 242, Elemental Analysis C, 74.36, H, 5.82, O, 19.81.

*Compounds (* ***iv (a*** *–* ***d)***). A solution of ester (23.6 g, 0.1 mol) and AlCl_3_ (13.3 g, 0.1 mol) was refluxed at 150°C till completion of reaction (as monitored by TLC). The reaction mixture was cooled, and then 5% cooled aqueous HCl was added till all the excess AlCl_3_ neutralized. The reaction mixture was extracted with ethyl acetate and the organic layer was collected, dried over Na_2_SO_4_, and concentrated over vacuum. The residue was chromatographed to obtain the pure compound. Yield:- 12%, (**iv-a**):- ^1^H NMR- (300 MHz, CDCl_3_): *δ* 3.79 (s, 3H, -CH_3_), 4.24 (s, 2H, -CH_2_), 6.90 (m, 3H, -Ar-H), 6.99 (s, 1H, -Ar-H), 7.18 (d, *J* = 8.4 Hz, 2H, -Ar-H), 7.46 (t, *J* = 7.5 Hz, 1H, -Ar-H), 7.86 (d, *J* = 6.9 Hz, 1H, -Ar-H); ^13^C NMR- (75 MHz, CDCl_3_): *δ* 44.231, 55.234, 114.219, 118.933, 125.790, 130.390, 136.463, 158.724, 162.870, 204.185; FTIR (KBr, cm^−1^): 3448, 2914, 2836, 1633, 1504, 1445, 1344, 1248, 844, 791, 751; *m*/*z* 242, Elemental Analysis C, 74.36, H, 5.82, O, 19.81 Figure S2(a, b&c); (**iv-b**):- ^1^H NMR- (300 MHz, CDCl_3_): 4.23 (s, 2H, -CH_2_), 6.806 (d, *J* = 8.4 Hz, 2H, -Ar-H), 6.886 (d, *J* = 7.5 Hz, 1H, -Ar-H), 6.960 (t, *J* = 8.4 Hz, 1H, -Ar-H), 7.132 (d, *J*=8.4 Hz, 2H, -Ar-H), 7.466 (t, *J* = 7.2 Hz, 1H, -Ar-H), 7.852 (d, *J* = 7.8 Hz, 1H, Ar-H); FTIR (KBr, cm^−1^): 3447, 3045, 2909, 1635, 1515, 1483, 1443, 1341, 847, 798, 754 (Figures S3(a) & S3(b)); *m*/*z* 328, Elemental Analysis C, 80.47, H, 4.91, O, 14.62. 

*General Procedure for Compounds (* ***vi (a*** *–* ***h)***)

*Compounds (* ***vi (a*** *-* ***h)***). To a solution of ortho substituted phenol (236 mg, 1 mmol) and K_2_CO_3_ (690 mg, 5 mmol) in dry acetone (25 mL) was added phenyl acetyl chloride (308 mg, 2 mmol) dropwise. The reaction mixture was refluxed at 100°C for 7 h. After the reaction was completed (as monitored by TLC), the reaction mixture was cooled, filtered, and concentrated. The residue was chromatographed to obtain the pure compound with 20% ethyl acetate-hexane. Yield:- 70%.

#### 4.1.2. Procedure for Scheme 2

*Compound (* ***ix*** *-* ***e***). To a solution of 4-methoxy phenyl acetic acid (166 mg, 1 mmol) in dry acetone (10 mL) was added BF_3_·Et_2_O (0.4 mL, 3 mmol) at 0°C. After 30 minutes, we added phenol (0.1 mL, 1 mmol) and refluxed it till the reaction was completed as monitored by TLC. Then we filtered the reaction mixture and evaporated the solvent in vacuum. White solid was obtained, recrystallized from ethanol. Yield:- 80%, m.p. 65°C, ^1^H NMR- (300 MHz, CDCl_3_): *δ* 3.79 (s, 3H, -CH_3_), 4.24 (s, 2H, -CH_2_), 6.90 (m, 3H, -Ar-H), 6.99 (s, 1H, -Ar-H), 7.18 (d, *J* = 8.4 Hz, 2H, -Ar-H), 7.46 (t, *J* = 7.5 Hz, 1H, -Ar-H), 7.86 (d, *J* = 6.9 Hz, 1H, -Ar-H); FTIR (KBr, cm^−1^): 3448, 2914, 2836, 1633, 1504, 1445, 1344, 1248, 844, 791, 751. (Figure S2(a, b & c). 

*Compound (* ***vi***-***e***). Phenyl acetyl chloride (0.13 mL, 1 mmol) was added to a solution of (***ix-e***) (242 mg, 1 mmol) in dry acetone and K_2_CO_3_ (552 mg, 4 mmol) and refluxed for 6 h. Then the reaction mixture was filtered and concentrated in vacuum. The obtained crude was recrystallized from ethanol to obtain the pure product. Yield:- 75%.

*Procedure for Compound (* ***vi*** *-* ***i***). Acetic anhydride (920 mg, 1 mL) was added to a solution of (**vi-a**) (328 mg, 1 mmol) and pyridine (0.25 mL, 9 mmol) and refluxed under nitrogen atmosphere for 6 h at 90°C. After the reaction was completed (as monitored by TLC), solvent was removed under vacuum. The residue was washed with saturated Na_2_HCO_3_ until excess pyridine was removed and then it was washed with aqueous HCl and finally with saturated brine solution and dried and chromatographed with 20% ethyl acetate-hexane. Yield: 90%, m.p. 160°C. 

*Analytical Data for Compounds (* ***vi (a*** *–* ***i)***)

(***vi-a***). ^1^H NMR- (300 MHz, CDCl_3_): *δ* 4.03 (s, 2H, -CH_2_), 6.717 (d, *J* = 8.4 Hz, 2H, Ar-H), 6.932 (d, *J* = 8.1 Hz, 2H, Ar-H), 7.170 (t, *J* = 7.5 Hz, 1H, Ar-H), 7.273 (d, *J* = 8.4 Hz, 2H, Ar-H), 7.384 (d, *J* = 7.5 Hz, 4H, Ar-H), 7.459 (m, 2H, Ar-H); FTIR (KBr, cm^−1^): 3484, 3433, 3059, 2931, 1707, 1604, 1564, 1513, 1446, 1267, 1173, 828, 750 Figure S5(a, b & c); *m*/*z* 342.13, Elemental Analysis C, 80.68, H, 5.30, O, 14.02; (**vi-b**):- ^1^H NMR- (300 MHz, CDCl_3_): *δ* 3.01 (s, 3H, -CH_3_), 4.05 (s, 2H, -CH_2_), 6.722 (d, *J* = 8.1 Hz, 2H, Ar-H), 6.937 (d, *J* = 7.2 Hz, 2H, Ar-H), 7.179 (t, *J* = 7.8 Hz, 1H, Ar-H), 7.266 (d, *J* = 8.1 Hz, 2H, Ar-H), 7.377 (d, *J* = 7.8 Hz, 4H, Ar-H), 7.450 (m, 2H, Ar-H); ^13^C NMR (75 MHz, CDCl_3_): *δ* 22.9, 40.2, 115.6, 122.3, 125.2, 126.3, 126.8, 128.0, 129.5, 130.5, 130.9, 132.0, 137.0, 145.1, 150.8, 155.2, 162.1; (**vi-c**):- ^1^H NMR- (300 MHz, CDCl_3_): *δ* 3.75 (s, 3H, -CH_3_), 4.03 (s, 2H, -CH_2_), 6.717 (d, *J* = 8.4 Hz, 2H, Ar-H), 6.932 (d, *J* = 8.1 Hz, 2H, Ar-H), 7.132 (d, *J* = 7.5 Hz, 2H, Ar-H), 7.363 (d, *J* = 7.5 Hz, 2H, Ar-H), 7.459 (m, 4H, Ar-H); ^13^C NMR (75 MHz, CDCl_3_): *δ* 40.1, 56.2, 114.0, 121.3, 122, 125.2, 127.2, 127.8, 128.1, 130.3, 130.6, 144.0, 150.8, 154.3, 161.2, 162.0; (**vi-d**):- ^1^H NMR- (300 MHz, CDCl_3_): *δ* 1.30 (s, 3H, -CH_3_), 3.51 (s, 2H, -CH_2_), 4.04 (s, 2H, -CH_2_), 6.721 (d, *J* = 8.1 Hz, 2H, Ar-H), 6.938 (d, *J* = 7.2 Hz, 2H, Ar-H), 7.202 (d, *J* = 7.5 Hz, 2H, Ar-H), 7.370 (d, *J* = 7.5 Hz, 2H, Ar-H), 7.459 (m, 4H, Ar-H); ^13^C NMR (75 MHz, CDCl_3_): *δ* 18.1, 28.6, 40.1, 115.6, 121.3, 122, 125.2, 126.4, 126.9, 128.0, 128.4, 130.5, 130.8, 132.3, 139.8, 145.0, 150.9, 155.0, 162.1; (**vi-e**):- ^1^H NMR- (300 MHz, CDCl_3_): *δ* 3.75 (s, 3H, -OCH_3_), 4.04 (s, 2H, -CH_2_), 6.79 (d, *J* = 8.4 Hz, 2H, Ar-H), 6.98 (d, *J* = 8.4 Hz, 2H, Ar-H), 7.15 (t, *J* = 7.5 Hz, 1H, Ar-H), 7.30 (s, 2H, -Ar-H), 7.37 (s, 4H, -Ar-H), 7.49 (q, *J* = 8.1 Hz, 2H, -Ar-H); ^13^C NMR (75 MHz, CDCl_3_): *δ* 34.74, 55.19, 114.19, 116.93, 119.60, 124.22, 126.40, 128.38, 128.47, 128.83, 129.53, 129.79, 131.19, 134.05, 149.06, 153.13, 158.18, 161.22; FTIR (KBr, cm^−1^): 3075, 2928, 2857, 1707, 1509, 1445, 1384, 1241, 1121, 836, 798; *m*/*z* 342.13, Elemental Analysis C, 80.68, H, 5.30, O, 14.02, Figure S6(a, b, c & d); (**vi-f**):- ^1^H NMR (300 MHz, CDCl_3_): *δ* 2.36 (s, 3H, -CH_3_), 3.75 (s, 3H, -OCH_3_), 4.04 (s, 2H, -CH_2_), 6.79 (d, *J* = 8.4 Hz, 2H, Ar-H), 6.98 (d, *J* = 8.4 Hz, 2H, Ar-H), 7.10 (s, 2H, -Ar-H), 7.29 (s, 4H, -Ar-H), 7.49 (q, *J* = 8.1 Hz, 2H, -Ar-H); ^13^C NMR (75 MHz, CDCl_3_): *δ* 21.0, 40.0, 56.4, 114.1, 121.6, 1222.0, 125.5, 126.1, 126.8, 128.1, 128.4, 129.3, 130.1, 130.6, 132.2, 137.2, 145.1, 150.9, 160.3, 162.0; (**vi-g**):- ^1^H NMR (300 MHz, CDCl_3_): *δ* 3.75 (s, 3H, -OCH_3_), 4.04 (s, 2H, -CH_2_), 6.67 (d, *J* = 8.4 Hz, 2H, Ar-H), 6.88 (d, *J* = 8.4 Hz, 2H, Ar-H), 7.15 (s, 2H, -Ar-H), 7.29 (s, 4H, -Ar-H), 7.45 (q, *J* = 8.1 Hz, 2H, -Ar-H); ^13^C NMR (75 MHz, CDCl_3_): *δ* 40.0, 56.4, 114.0, 115.6, 121.6, 121.8, 125.7, 126.9, 127.6, 127.8, 128.1, 130.0, 130.4, 144.0, 150.9, 156.7, 159.6, 162.1; *m*/*z* 358, C, 77.08, H, 5.06, O, 17.86; (**vi-h**):- ^1^H NMR (300 MHz, CDCl_3_): *δ* 1.30 (s, 3H, -CH_3_), 3.51 (s, 2H, -CH_2_), 3.75 (s, 3H, -OCH_3_), 4.04 (s, 2H, -CH_2_), 6.67 (d, *J* = 8.4 Hz, 2H, Ar-H), 6.88 (d, *J* = 8.4 Hz, 2H, Ar-H), 7.15 (s, 2H, -Ar-H), 7.29 (s, 4H, -Ar-H), 7.45 (q, *J* = 8.1 Hz, 2H, -Ar-H); ^13^C NMR (75 MHz, CDCl_3_): *δ* 18.1, 29.6, 40.1, 56.0, 114.2, 121.4, 122.3, 125.2, 126.3, 127.8, 128.4, 130.0, 130.2, 132.1, 140.0, 144.0, 151.1, 159.2, 162.0. 

(***vi*** *-* ***i***). ^1^H NMR- (300 MHz, CDCl_3_): *δ* 2.29 (s, 3H, CH_3_), 4.15 (s, 2H, CH_2_), 7.01 (d, *J* = 6.69 Hz, 2H, Ar-H), 7.10 (d, *J* = 8.64 Hz, 2H, Ar-H), 7.20 (t, *J* = 1.44 Hz, 1H, Ar-H), 7.30 (m, 2H, Ar-H), 7.43 (m, 4H, Ar-H), 7.53 (m, 2H, Ar-H) (Figure S7); *m*/*z* 370.12, Elemental Analysis C, 77.82, H, 4.90, O, 17.28; *m*/*z* 370, C, 81.06, H, 5.99, O, 12.96. 

*Note*. Crystallographic information is given in the supporting file with details of refinement and other structural parameters.

## Supplementary Material

Supplementary Material provides the Instrumentation details, crystal structure & refinement, NMR (^1^HNMR and ^13^CNMR), FTIR and Mass spectra of pro SERMs.

## Figures and Tables

**Table 1:** Bond lengths and bond angles of (**vi-e**) have been demonstrated.

S. number	Atoms	Bond lengths	Atoms	Bond angles
1	O3-C20	1.3772(1)	C20-O3-C23	117.76
2	O3-C23	1.3963(1)	C8-O1-C7	121.71
3	O1-C8	1.3848(1)	O3-C20-C21	115.93
4	O1-C7	1.3722(1)	O1-C8-C1	115.40
5	C5-C6	1.3610(1)	O3-C20-C19	124.73
6	C6-C7	1.4647(1)	O1-C7-C6	117.78
7	O2-C7	1.2114(1)	C6-C7-O2	125.71

**Table 2:** Derivatives of 4-aryl-3-phenyl-coumarin-2-one and their yield (%) for Scheme 2.

S. number	Compound	R	R_2_	Time (h)	Yield^a^ (%)
1	(**vi-a**)	-OH	H	7	74
2	(**vi-b**)	-OH	-CH_3_	7	77
3	(**vi-c**)	-OH	-OCH_3_	6	80
4	(**vi-d**)	-OH	-C_2_H_5_	8	70
5	(**vi-e**)	-OCH_3_	H	7	75
6	(**vi-f**)	-OCH_3_	-CH_3_	8	79
7	(**vi-g**)	-OCH_3_	-OCH_3_	7	82
8	(**vi-h**)	-OCH_3_	-C_2_H_5_	7	80
9	(**vi-i**)	-OAc	H	6	90

^a^The reaction yield refers to product isolated through column chromatography.
