# Supplementary material for: BF3·Et2O Catalysed 4-Aryl-3-phenyl-benzopyrones, Pro-SERMs, and Their Characterization
Source: Adv Pharmacol Sci. 2015 Sep 1;2015:527159. doi: 10.1155/2015/527159 (PMC4569759; doi:10.1155/2015/527159)
Supplement: Supplementary file 1 — Supplementary Material provides the Instrumentation details, crystal structure & refinement, NMR (1HNMR and 13CNMR), FTIR and Mass spectra of pro SERMs. [file 527159.f1.doc]

Supporting Information

**BF_3_.Et_2_O catalyzed synthesis of 4-aryl-3-phenyl-benzopyrones, *pro* SERMs and their characterization**

*Ambika Srivastava, Pooja Singh and Rajesh Kumar[[1]](#footnote-2)**

Department of Chemistry, Centre of Advanced Study, Faculty of Science, Banaras Hindu University, Varanasi-221005, U.P. India, E-mail: [rkr_bhu@yahoo.com](mailto:rkr_bhu@yahoo.com)

**Experimental Section**

**Materials and Methods**

All starting materials were commercially available and used as received without further purification. Commercially available acetone and benzene were further purified and dried following the known procedure. Thin-layer chromatography (TLC) was performed using silica gel 60 F254 precoated plates. Infrared (FTIR) spectra are measured in KBr, and wavelengths (ν) are reported in cm^-1^, ^1^H and ^13^C NMR spectra were recorded on NMR spectrometers operating at 300 and 75.5MHz, respectively. Chemical shifts (δ) are given in parts per million (ppm) using the residue solvent peaks as reference relative to TMS. J values are given in Hz. Mass spectra were recorded using electro spray ionization (ESI) mass spectrometry. The melting points are uncorrected.

**Crystal structure determination and reﬁnement**

Data for the structure (vi) where R=OCH_3_ and R_1_ =H was obtained at 293(2) K, on Oxford Gemini diffractometers, both equipped with SMART 6000 CCD software using graphite mono-chromated Mo Ka (k = 0.71073 Å ) radiation (Table 2). The structures were solved by direct methods (SHELXS-97) and reﬁned against all data by full matrix least-square on F^2^ using anisotropic displacement parameters for all non-hydrogen atoms. All hydrogen atoms were included in the reﬁnement at geometrically ideal position and reﬁned with a riding model ^[1]^. The MERCURY and encipher 1.3 packages were used for molecular graphics ^[2^, ^3]^. Molecular structures were generated by use of the ORTEP-3 for windows program ^[4]^. Crystallographic data and reﬁnement details for the structural analysis are summarized in Table-S1 and selected bond lengths and bond angles are given in Tables-2 (main text).

**References**

1. Sheldrick, G. M. *Acta Cryst*. A. **2008**, 64, 112.
2. Bruno, I. J.; Cole, J.C.; Edgington, P. R.; Kessler, M.; Macrae, C. F.; McCabe, P.; Pearson, J.; Taylor, R. *Acta Crystallogr Sect* B. **2002**, 58, 389. doi:10.1107/S01087 68102003324
3. Brandenburg, K.; Putz, H. **2004** Diamond *version* 3.0. University of Bonn, Germany
4. Farrugia, L. J. *J.* *Appl Crystallogr*. **1997**, 30, 565. doi:10.1107/S0021889897003117

**Table S1 Crystallographic data and structure refinement for structure (vi (e)**)

| S.No. | Compound (vi-e) | Where R=OCH_3_, R_1_=H |
| --- | --- | --- |
|  | CCDC no. | 797350 |
| 1. | Empirical Formula | C_23_H_18_O_3_ |
| 2. | Formula weight | 342.37 |
| 3. | T(K) | 293(2) |
| 4. | λ (Mo Kα)(Ǻ) | 0.71073 |
| 5. | Crystal system | Orthorhombic |
| 6. | Space group | Pbca |
| 7. | a (Ǻ) | 12.7577(14) |
| 8. | b (Ǻ) | 13.3400(15) |
| 9. | c (Ǻ) | 20.582(3) |
| 10. | α(˚) | 90 |
| 11. | β(˚) | 90 |
| 12. | γ(˚) | 90 |
| 13. | V (Ǻ^3^) | 3502.8(7) |
| 14. | Z | 8 |
| 15. | ρ_calcd_ (mg/m^3^) | 1.298 |
| 16. | Crystal size (mm^3^) | 0.27×0.25×0.23 |
| 17. | F(000) | 1440 |
| 18. | μ(mm^-1^) | 0.085 |
| 19. | θ range for data collection (˚) | 3.21 – 29.14 |
| 20. | Index ranges | -14 ≤ h ≤ 17  -18 ≤ k ≤ 16  -24 ≤ l ≤ 25 |
| 21. | No. of reﬂections collected | 4720 |
| 22. | No. of independent reﬂections | 884 |
| 23. | Number of data/restrains/parameters | 884/0/249 |
| 24. | Goodness-of-ﬁt on F^2^ | 0.771 |
| 25. | R_1_^α^, wR_2_^b^ [I>2r(I)] | 0.3621, 0.0877 |
| 26. | R_1_^α^, wR_2_^b^ (all data) | 0.1512, 0.0856 |
| 27. | Largest difference in peak and hole(e Ǻ^–3^) | 0.181, -0.188 |


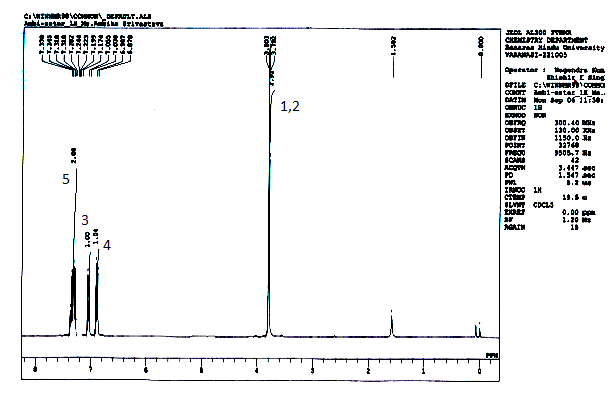


Fig.S1(a) ^1^H NMR of (iii)


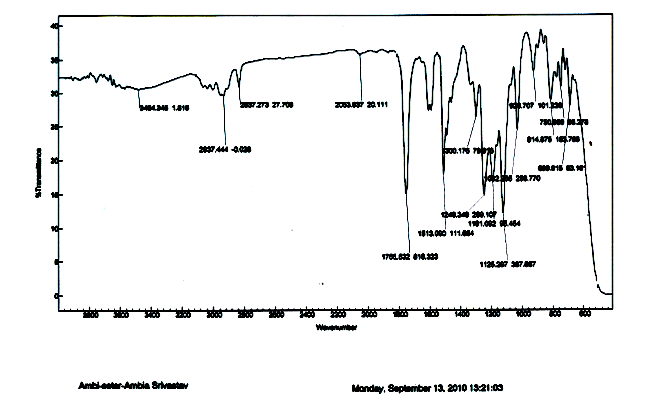
Fig.S1(b) FTIR of (iii)


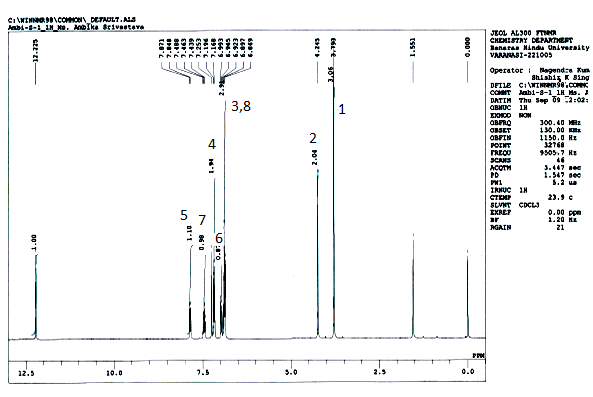


Fig.S2(a) ^1^H NMR of (iv)a


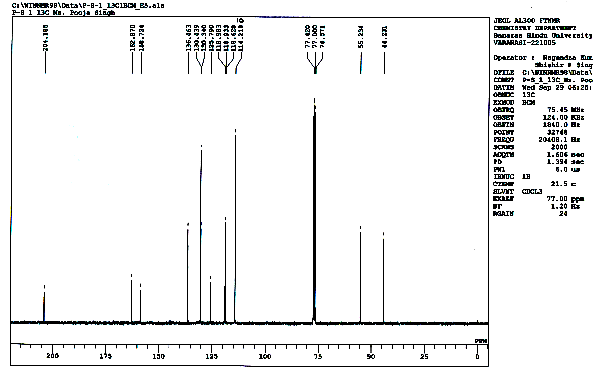


Fig.S2(b) ^13^C NMR of (iv)a


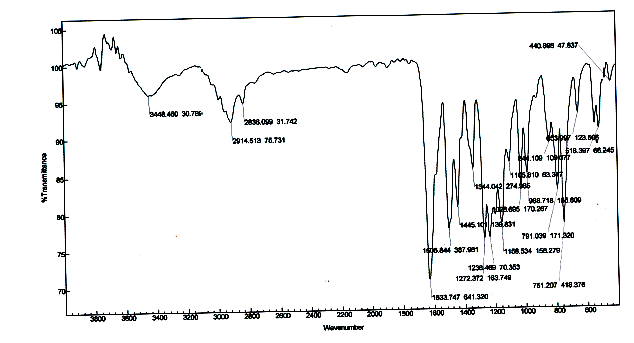
Fig.S2(c) FTIR of (iv)a


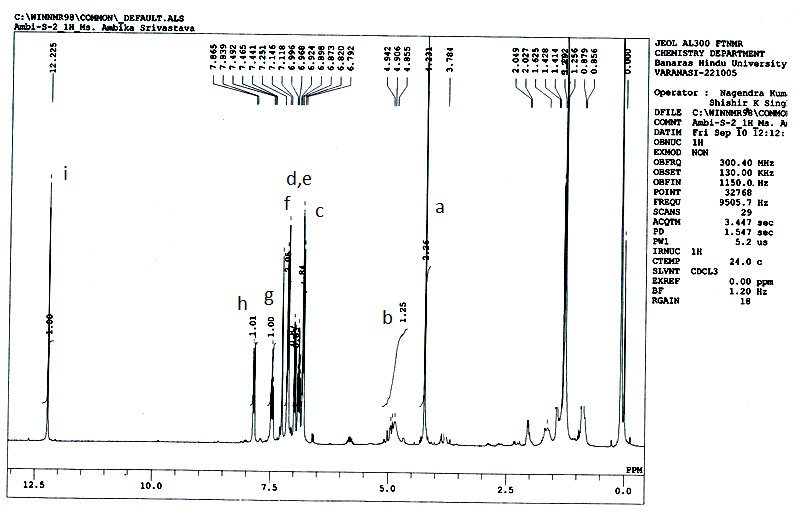


Fig.S3(a) ^1^H NMR of (iv)b


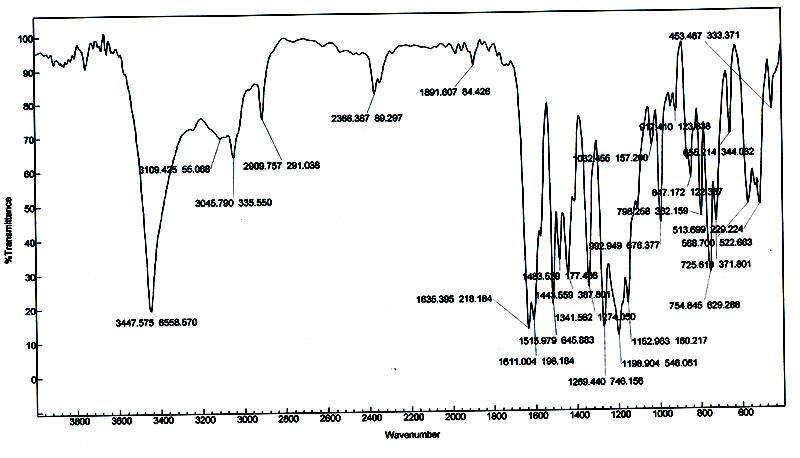
Fig.S3(b) FTIR of (iv)b


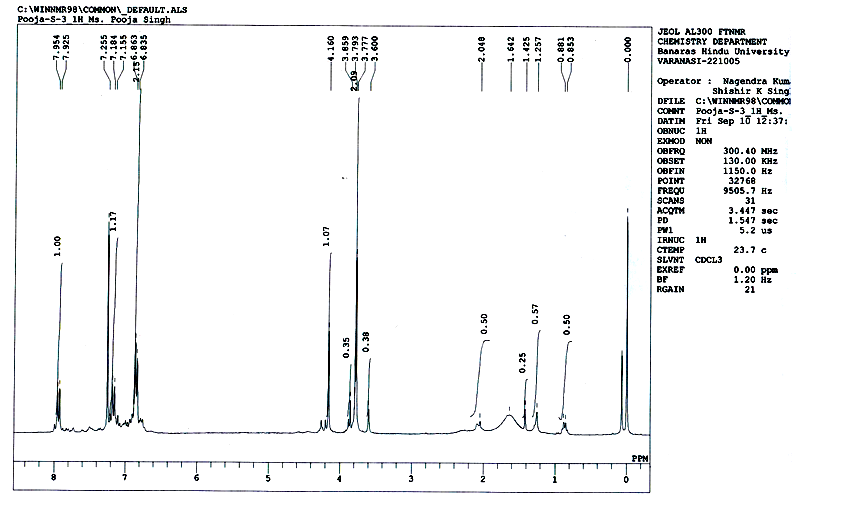


Fig.S4(a) ^1^H NMR of (iv)c


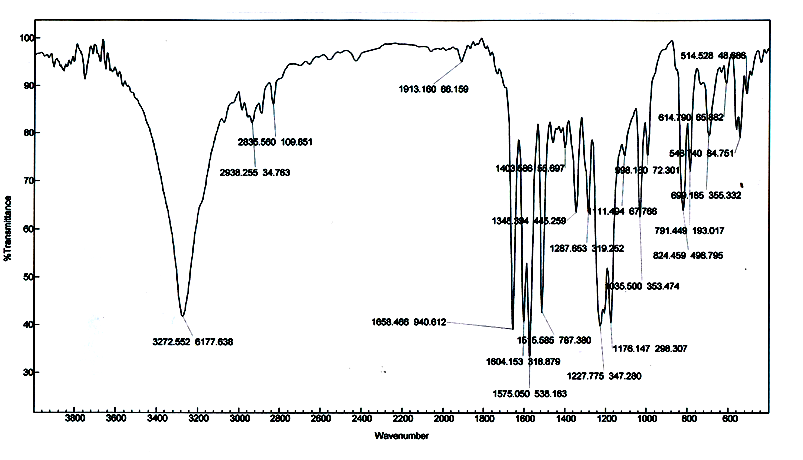


Fig.S4(b) FTIR of (iv)c


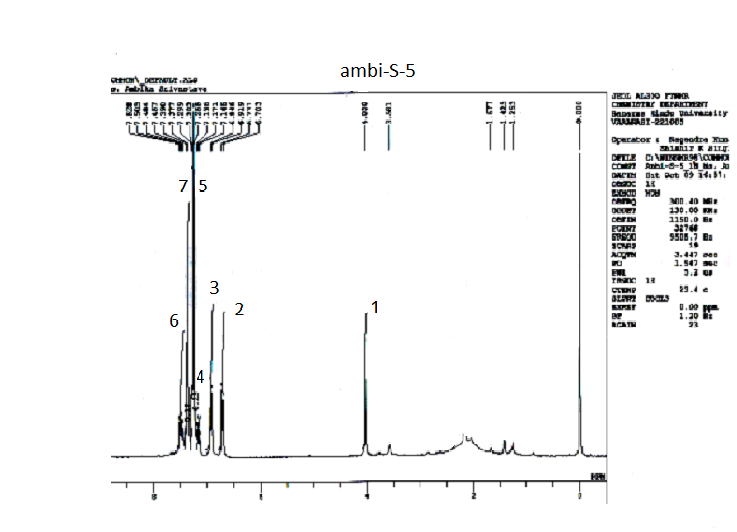


Fig.S5(a) ^1^H NMR of (vi)a


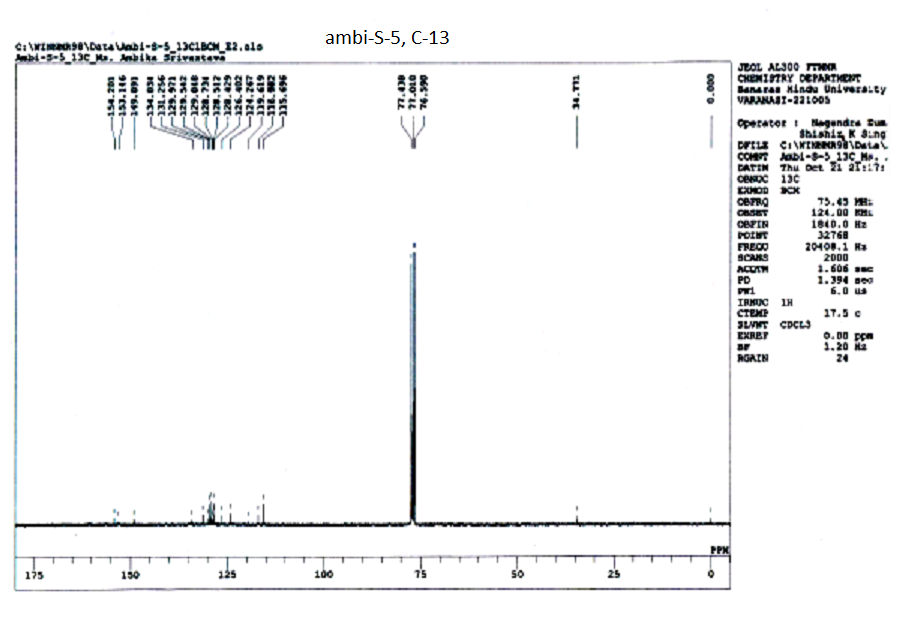


Fig.S5(b) ^13^C NMR of (vi)a


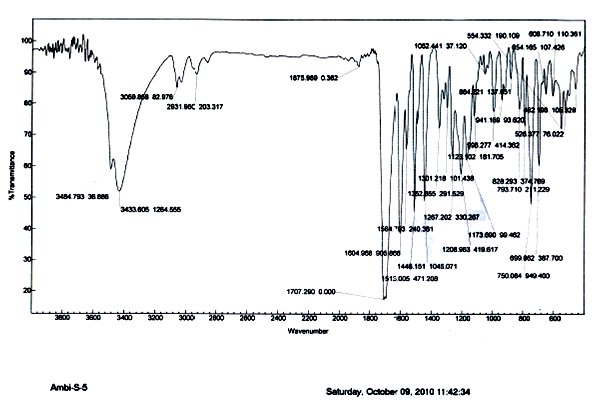


Fig.S5(c) FTIR of (vi)a


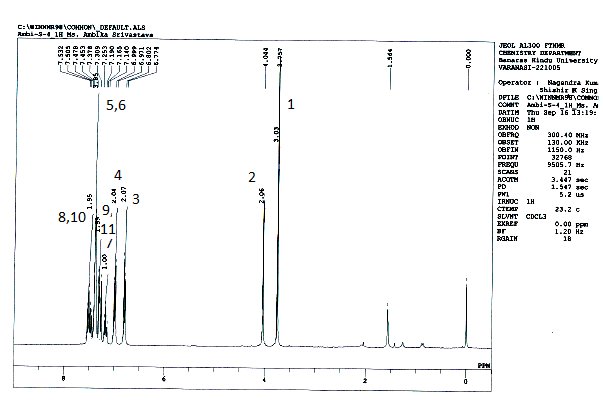


Fig. S6 (a) ^1^H NMR of (vi)e


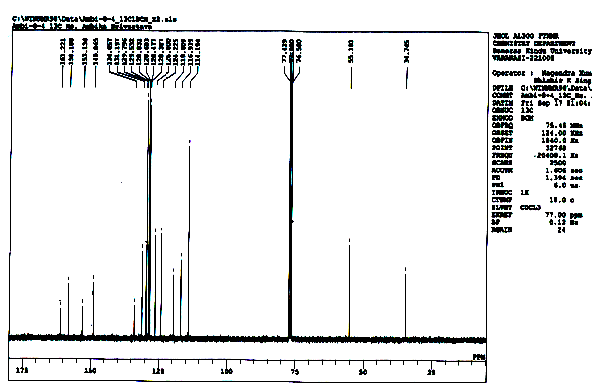


Fig. S6(b) ^13^C NMR of (vi)e


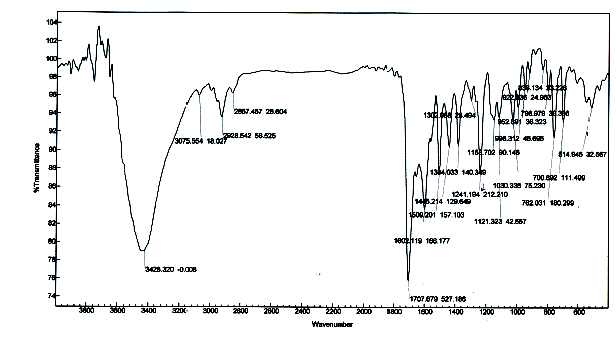


Fig. S6(c) FTIR of (vi) e


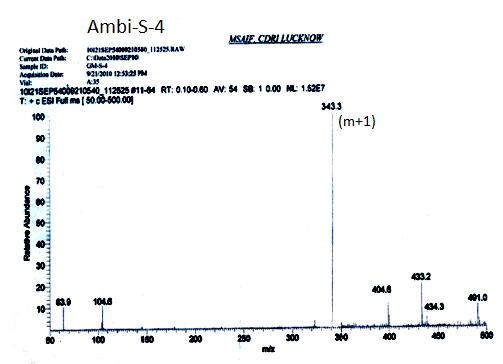


Fig. S6(d) Mass spectra of (vi) e


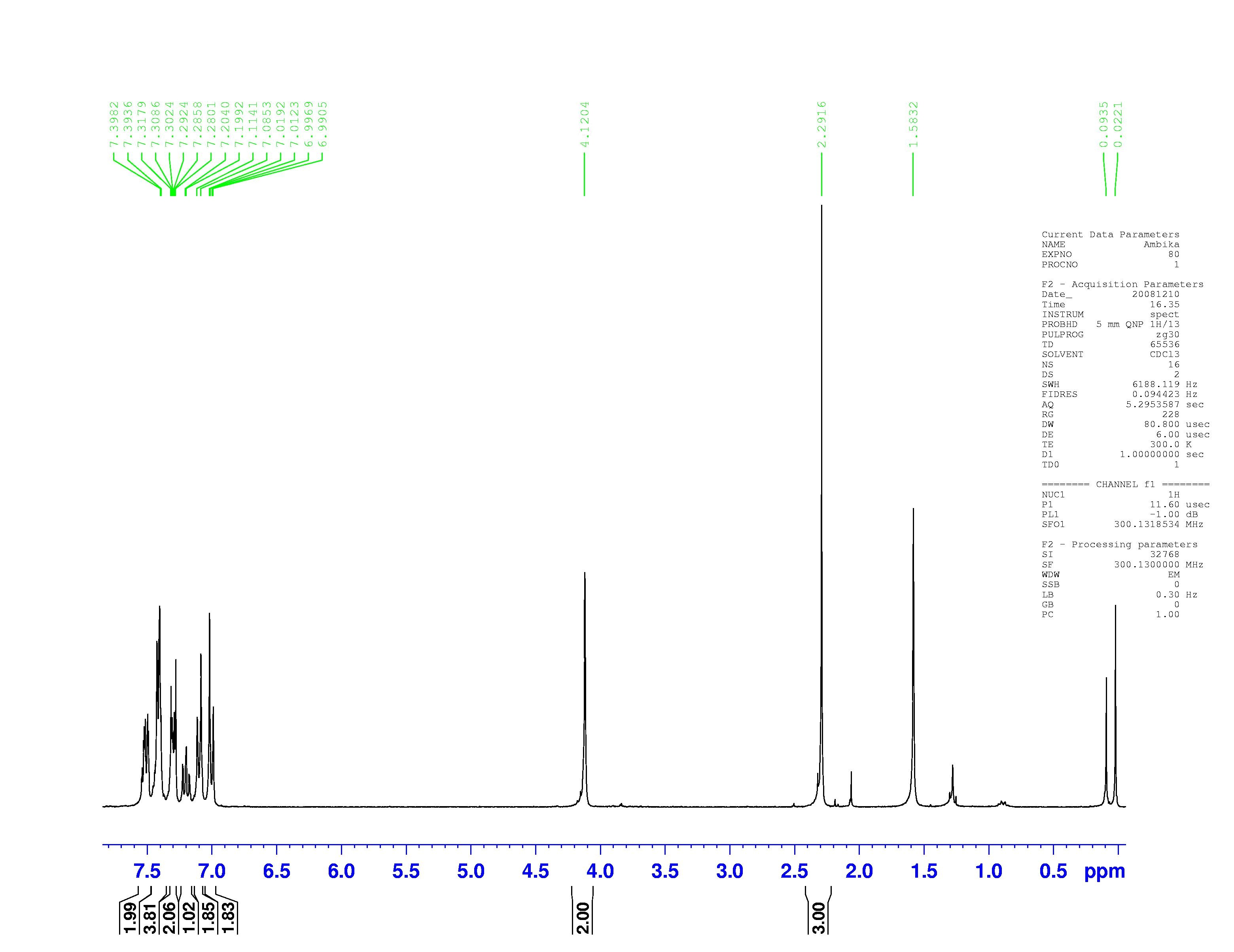


Fig. S7. ^1^H NMR of (vi) i

1. ^^ To whom correspondence should be addressed: Department of Chemistry, Centre of Advanced Study, Faculty of Science, Banaras Hindu University, Varanasi-221005, U.P. India, E-mail: [rkr_bhu@yahoo.com](mailto:rkr_bhu@yahoo.com), Phone No. : +91-542-6702501, Fax No. : +91-542-2368174. [↑](#footnote-ref-2)
